# Supplementary material for: Hybrid Models and Biological Model Reduction with PyDSTool
Source: PLoS Comput Biol. 2012 Aug 9;8(8):e1002628. doi: 10.1371/journal.pcbi.1002628 (PMC3415397; doi:10.1371/journal.pcbi.1002628)
Supplement: Text S4 — Complete source code for the PyDSTool package (version 0.88.120504). Includes API documentation and help files linking to web pages. This file is identical to the current public release on Sourceforge.net. (ZIP) [file pcbi.1002628.s004.zip › PyDSTool/html/PyDSTool.Toolbox.optimizers.optimizer.optimizer.Optimizer-class.html]

xml version="1.0" encoding="ascii"?


PyDSTool.Toolbox.optimizers.optimizer.optimizer.Optimizer


| Home | Trees | Indices | Help | PyDSTool |
| --- | --- | --- | --- | --- |

Package PyDSTool ::
Package Toolbox ::
Package optimizers ::
Package optimizer ::
Module optimizer ::
Class Optimizer

[hide private]

[frames] | no frames]

# Class Optimizer

source code

```
object --+
         |
        Optimizer
```

Known Subclasses:
:   - standard\_optimizer.StandardOptimizer
    - , standard\_optimizer\_modifying.StandardOptimizerModifying

---

The simple optimizer class This class lacks some intel that must be
populated/implemented in the subclasses :

- currentValues is a list of the 2 last cost values computed with the
  function
- currentParameters is a list of the 2 last parameters used for the
  computation of currentValues
- the iterate function that does the real iteration loop


|  |  |
| --- | --- |
| Instance Methods | [hide private] |

|  |  |
| --- | --- |
| \_\_init\_\_(self, \*\*kwargs)  Initialization of the optimizer, saves the function and the criterion to use Needs to have : | source code |

|  |  |
| --- | --- |
| optimize(self)  Does the optimization, call iterate and returns the optimal set of parameters | source code |

|  |  |
| --- | --- |
| recordHistory(self, \*\*kwargs)  Function that does nothing, called for saving parameters in the iteration loop, if needed | source code |

|  |  |
| --- | --- |
| iterate(self)  Does one iteration of the optimization Present here for readability | source code |

|  |  |
| --- | --- |
| check\_arguments(self)  Checks if the given arguments are correct | source code |

**Inherited from `object`**:
`__delattr__`,
`__getattribute__`,
`__hash__`,
`__new__`,
`__reduce__`,
`__reduce_ex__`,
`__repr__`,
`__setattr__`,
`__str__`


|  |  |
| --- | --- |
| Properties | [hide private] |

**Inherited from `object`**:
`__class__`


|  |  |
| --- | --- |
| Method Details | [hide private] |

|  |  |
| --- | --- |
| \_\_init\_\_(self, \*\*kwargs)  *(Constructor)* | source code |

Initialization of the optimizer, saves the function and the criterion
to use Needs to have :

- a function to optimize (function)
- a criterion to stop the optimization (criterion)

Can have :

- a step modifier, a factor to modulate the step (stepSize = 1.)
- a recorder that will be called with the factors used in one iteration
  step (record = self.recordHistory)

Overrides: object.\_\_init\_\_

  


| Home | Trees | Indices | Help | PyDSTool |
| --- | --- | --- | --- | --- |

|  |  |
| --- | --- |
| Generated by Epydoc 3.0.1 on Fri May 4 15:24:09 2012 | http://epydoc.sourceforge.net |
